# Chlorpyrifos removal: Nb/boron-doped diamond anode coupled with solid polymer electrolyte and ultrasound irradiation

**DOI:** 10.1007/s40201-020-00555-z

**Published:** 2020-10-09

**Authors:** Andrea Luca Tasca, Davide Clematis, Marco Panizza, Sandra Vitolo, Monica Puccini

**Affiliations:** 1 Department of Civil and Industrial Engineering, University of Pisa, Largo Lucio Lazzarino, Pisa, 56122 Italy; 2 Department of Civil, Chemical and Environmental Engineering, University of Genoa, Via Opera Pia 15, Genoa, 16145 Italy

**Keywords:** Boron-doped diamond, Solid polymer electrolyte, By-products, Water treatment, Anodic oxidation, Sonication.

## Abstract

Chlorpyrifos is an organophosphorus insecticide, acaricide and miticide used worldwide for the control of soil-borne insect pests. It must be considered as a substance of growing concern, given its use, toxicity, environmental occurrence, and potential for regional to long-range atmospheric transport. Considering the incomplete removal attained by conventional water treatment processes, we investigated the efficiency of electrolytic radicals production and sonoelectrolysis on the degradation of the pesticide. The treatment has been conducted in a novel electrochemical reactor, equipped with a boron-doped diamond anode and a solid polymer electrolyte (SPE). Different current intensity and times have been tested and coupled with sonication at 40 kHz. Up to 69% of chlorpyrifos was completely removed in 10 min by electrolysis operated at 0.1 mA, while 12.5% and 5.4% was converted into the treatment intermediates 3,5,6-trichloro-2-pyridinol (TCP) and diethyl (3,5,6-trichloropyridin-2-yl) phosphate, respectively. Ultrasound irradiation did not enhance the removal efficiency, likely due to mass transport limitations, while the energy consumption increased from 8.68∙10^− 6^ to 9.34∙10^− 4^ kWh µg^− 1^ removed. Further research is encouraged, given the promising processing by the SPE technology of low conductivity solutions, as pharmaceuticals streams, as well as the potential for water and in-situ groundwater remediation from different emerging pollutants as phytosanitary and personal care products.

## Introduction

Chlorpyrifos (CP) is an organophosphorus insecticide, acaricide and miticide effective against a broad spectrum of insect pests. CP is a neurotoxic compound. The inhibition of the enzyme acetylcholinesterase in the central and peripheral nervous systems leads to the breakdown of the neurotransmitter acetylcholine, overstimulating muscarinic and nicotinic receptors (Zhao et al. 2006). This pesticide is part of the Endocrine Disrupting Chemicals (EDCs), a class of molecules with proven effects on male and female reproduction, breast and prostate cancer, neuroendocrinology, thyroid, metabolism and cardiovascular endocrinology (Tasca and Fletcher, 2017). Human and wildlife exposure has been linked to atmospheric, water and soil resources contamination, as well as to food intake (Salamzadeh et al. 2018).

Inhibitory effects on soil microbial functional diversity have been reported (Fang et al. 2009). CP levels in Argentinian irrigation channels have been associated to a significant reduction of macroinvertebrate abundance and taxon richness (Macchi et al. 2018). Freshwater crabs Barytelphusa guerini exposed to CP exhibited restlessness, escape behavior, increased scaphognathite activity, topsy-turving response and increased oxygen consumption. The LC_50_ values were 47.97 and 38.81 µg L^− 1^ after 24 and 96 h, respectively (Srivastava et al. 2013). The lethal times LT_50_ and LT_90_ for orally treated Apis mellifera workers were calculated as 171.1 and 3.2 h, 839.7 and 11.7 h at 0.001 and 100 ppm, respectively. The lethal concentrations on honeybee workers LC_50_ and LC_90_ ranged from 170.6 to 0.01 ppm and from 2855.2 to 1.4 ppm after 3 and 120 h of treatment, respectively. CP application in cotton fields resulted in the death of 39.7% of the bee workers, and in a significant decrease of bee foraging activities (Razik 2019). An increased risk of oral cytotoxicity has been demonstrated by degenerative changes of the tongue and lingual glands of Wistar rats orally treated with 3.375 to 13.5 mg kg^− 1^ d^− 1^ (1/40 to 1/10 of LD_50_) (El-Sayed et al. 2018).

Human dietary exposure to CP has been linked to dysbiosis of the intestinal microbiota (Condette et al. 2015; Réquilé et al. 2018). Chronic health effects, as developmental and neurobehavioral anomalies, have been reported (Atabila et al. 2018). The WHO set acute and chronic guideline values of CP to 100 and 10 µg kg^− 1^ d^− 1^, respectively (WHO 2009), while guideline values of 5 and 0.3 µg kg^− 1^ d^− 1^ have been established by the USEPA (Smegal, 2000).

The environmental behavior of CP is mainly governed by a high log *K*_OW_ value (4.7) and low water solubility (1.4 mg L^− 1^ at 25 °C). It is strongly adsorbed on soils and sediments, showing persistence under acidic pH and tendency for hydrolysis to 3,5,6-trichloro-2-pyridinol (TCP) in basic conditions (Báez et al. 2018; Smolen and Stone 1997). The half-life in soil was estimated as: 6.7, 33.9 and 119.6 days, during a field study carried out on soil treated with the × 1, × 2 and × 5 recommended dose rates. Temperature in soil and air ranged between − 1 and 11 °C, while 8 intense precipitation events (> 10 mm) occurred during the study. The adsorption coefficient *K*_*f*_ of the commercial formulation was 211.4 mg^1 − N^ L^N^ kg^− 1^, confirming a significant adsorption affinity (Papadopoulou et al. 2016).

CP was one of the two most detected pesticides in a pilot study to examine indoor (78.8%) and outdoor (39.3%) dust of 56 homes in Hualien County (Taiwan), suggesting that pesticide drift from agricultural areas to residential environments occurs (Hung et al. 2018). Moreover, notwithstanding half-life in air of 1.5 and 3 h at •OH concentrations of 1.5∙10^6^ and 0.7∙10^6^ molecules cm^3^, respectively, this pesticide is subject to long range transport (Mackay et al. 2014). Photolysis of the herbicide occurs on the surface of leaves and soils to form OCP, in which the sulfur atom is replaced by oxygen. Both CP and OCP are subject to volatilization (Mackay et al. 2014). The main removal pathway of CP in atmospheric environment is the reaction with hydroxyl radicals, with minor contributions of direct photolysis and reactions with ozone and nitrate radicals. CP disappearance in aquatic systems has been ascribed mainly to hydrolysis, photolysis, and microbial transformation (Giesy et al. 2014); half-lives of 73, 72, and 16 days have been estimated at 25 °C and pH 5, 7, and 9, respectively (Racke 1993).

Occurrence of this pesticide has been widely recorded. Concentrations up to 1.45 µg L^− 1^ have been measured in drainage channels of a 110-Ha section of an agricultural area near the Neuquén River (Argentina), with repeated exceedance of the LC_50_ of monitored macroinvertebrates (Macchi et al. 2018). The analysis of water samples collected from traditional wells, boreholes, and a lake in Burkina Faso between 2014 and 2016 showed that the threshold limit of 0.1 µg L^− 1^ has been exceeded two times in drinking water sources, while CP detection in water was systematically associated with a risk for the environment (Lehmann et al. 2018). CP concentration above 500 ng L^− 1^ has been recently recorded in a monitoring campaign of the river Nile (Dahshan et al. 2016). CP was the most detected compound in surface waters and sediment samples among 19 pesticides screened between 2012 and 2013 in Guangzhou, China. It has been detected in concentrations up to 414 ng L^− 1^ in surface water and 243 ng g_dw_^−1^ in sediment. Risk Quotients (RQs), expressed as the ratio between the measured environmental concentrations (MEC) and the predicted no effect concentrations (PNEC), showed that CP pose high ecological risk in environmental waters (Tang et al. 2019).

In 2011 an extensive survey on 50 pesticides was carried out in 16 WWTPs of Ebro, Guadalquivir, Jucar and Llobregat Rivers (Spain) (Campo et al. 2013). Higher concentrations in influents of the Ebro plants were found for CP (37 ng L^− 1^). The pesticide was detected also in the inlet streams of the remaining WWTPs (up to 108.68 ng L^− 1^). The layout of all the plants included screening, sand and fat-free/biological treatment (aeration, flocculation and settling); seven of them also included denitrification, while microfiltration, blending and deodorization were carried only by two WWTP. The removal efficiency of CP did not exceed the 75%, confirming that WWTPs may constitute a focal point of river contamination. Moreover, the mean CP content of the dehydrated sludge was 92.86 ng g^− 1^, revealing that the removal is partially attained only by transferring the pollutant into the sludge, which becomes a secondary source of pollution (Tasca et al. 2019b). Hence, new technologies are demanded.

The electrogeneration of hydroxyl radicals is one of the most efficient advanced oxidation processes. The reactivity of the radicals is linked to the interaction with the anode surface. Hence, Boron-Doped Diamond (BDD) electrodes yield to high mineralization rates (Kapałka et al. 2009), due to their very weak interaction. Hydroxyl radicals are produced by water electrolysis on the anode surface:

1 $$\text{H}_2\text{O}\rightarrow\bullet\text{OH}+\text{H}++ \ \text{e}^-$$

while the hydrogen evolution at the cathode has no effect on the mineralization of the contaminant:

2 $$2\text{H}_2\text{O}+2\text{e}^-\rightarrow\text{H}_2+2\text{OH}^-$$

The removal efficiency of anodic oxidation could be enhanced by ultrasound irradiation. Indeed, recently sonochemistry has shown promising application in Focused Ultrasound Surgery (Ebbini and Ter Haar, 2015; Mihcin and Melzer, 2018; Morchi et al. 2019), targeted drug delivery (Castle et al. 2013; Dromi et al. 2007; Ricotti et al. 2015), proteomics (Jorge et al. 2019, 2018; López-Ferrer et al. 2008) and water treatment (Guo et al. 2010; Homem and Santos, 2011; Kargar et al. 2012; Tasca et al. 2020; Villaroel et al. 2014; Villegas-Guzman et al. 2015). Sonochemical techniques are founded on the introduction of high-power ultrasound into the medium to be treated, alternating low- and high-pressure cycles. The bubbles formed during the low-pressure cycle grow to a critical size and collapse during a high pressure cycle, generating hot spots with singular conditions of pressure (∼1000 atm) and temperature (∼5000 K) (Adewuyi, 2001). Hydroxyl radicals are produced from water molecules and oxygen rupture and partially recombine to form hydrogen peroxide, with proven oxidation potential but lower than that of hydroxyl radicals (Ahmadi et al. 2020; Serna-Galvis et al. 2019; Yousefzadeh et al. 2014):

3 $${H}_{2}O \underrightarrow{\left)\right))} \bullet H+ \bullet OH$$

4 $${O}_{2} \underrightarrow{\left)\right))}2\bullet O$$

5 $${H}_{2}O+ \bullet O\to 2\bullet OH$$

6 $${O}_{2}+ \bullet H\to \bullet O+ \bullet OH$$

7 $$\bullet OH+ \bullet OH\to {H}_{2}{O}_{2}$$

Sonication was recently carried out successfully on a CP solution in an open stainless steel ultrasonic bath (Agarwal et al. 2016). However, oxidative pathways induced by ultrasound irradiation of water media are not accompanied by high mineralization ability (Rayaroth et al. 2016; Serna-Galvis et al. 2016), while coupling this processes with advanced oxidation techniques may promote the transformation of organic pollutants to CO_2_, water and inorganic ions (Zhang et al. 2011). At the best of our knowledge, no studies are available on the combined effect of ultrasounds and anodic oxidation on the degradation of chlorpyrifos, as there is still no information on the performance of Nb/BDD anode coupled with Solid Polymer Electrolyte (SPE) on the degradation of this pesticide. In this study we combined ultrasound irradiation with the electrochemical generation of ∙OH by Nb/BDD anode to remove CP from aqueous solutions. An ion exchange membrane was used as SPE to overcome the low conductivity of the treated media.

## Materials and methods

### Extraction and analysis

Fifty µg kg^− 1^ of O,O-Dimethyl O-(2,4,5-trichlorophenyl) phosphorothioate (Ronnel) were dissolved in 15 g of each sample, then 5 mL of a 1:1 mixture of cyclohexane/ethylacetate were used for the extraction, performed in triplicate by rotary evaporation. The so-obtained solution was then mixed with 300 mL of acetone and injected in a Shimadzu GC-MS-TQ8040, equipped with a crossbond diphenyl dimethyl polysiloxane SH-Rxi-5 ms column (45 m x 0,25 mm, 0.25 µm). Chromatographic conditions are detailed in Table 1. Helium was chosen as carrier gas (1.96 mL min^− 1^), injection was made in splitless mode, sampling time and purge flow were set at 1 min and 3 mL min^− 1^, respectively.

**Table 1:** Chromatographic ramp

Ramp	Temperature	Holding time
(°C/min)	(°C)	(min)
-	80	1
30	150	2
5	200	0
50	310	2.47

### Anodic oxidation and sonication

Anodic oxidation was performed in a single compartment electrochemical cell. The electric current was provided by an AMEL 2055 potentiostat/galvanostat. A Nb/BDD (DIACHEM®, Condias) and a Ti/RuO_2_ mesh (De Nora Industries) electrodes were used as anode and cathode, respectively. The size of the electrodes was: 3.5 cm × 7.5 cm, while the distance between them was fixed to 0.15 mm. A Nafion® N324 ion exchange membrane sandwiched between the electrodes was used as solid polymer electrolyte. Trials were conducted at 20 °C, under galvanostatic conditions and natural pH. Aqueous solutions (~ 0.30 dm^3^) of CP (initial concentration: 0.56 µg L^− 1^) were treated for 30 min, at current intensity of 0.1 and 0.5 A. Solutions were maintained mixed by a vertical stirrer, at a constant speed of 550 rpm. The ultrasonic device SONICA 2200 (Soltec, Italy) was used for ultrasound irradiation when sonication (40 kHz) was provided.

**Fig. 1:**
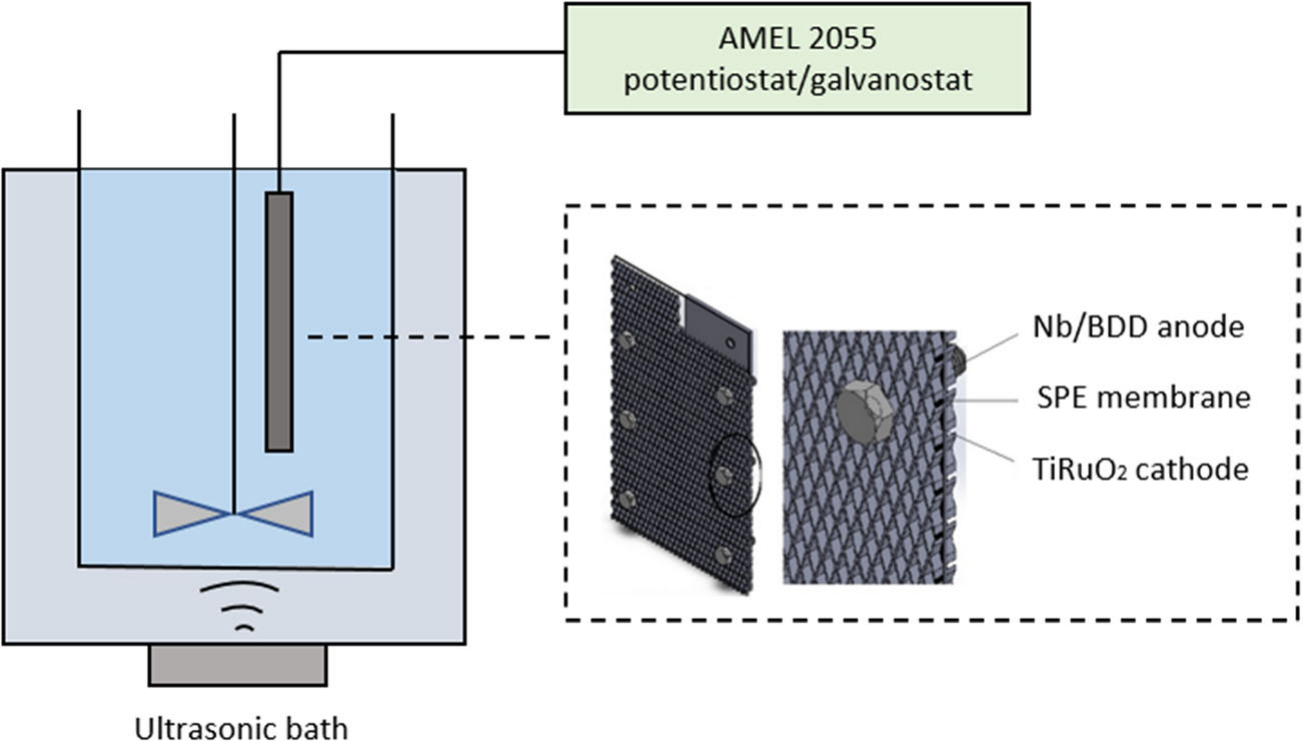
Sono-electrolytic cell

Experimental setup is shown in Fig. 1. Sampling was performed at defined intervals and aliquots were stored in the dark at 4 °C prior extraction and analysis. Prior each galvanostatic electrolysis assay, the electrodes were sonicated 30 min at current intensity of 1 A to remove any kind of impurity from their surface.

## Results and discussion

**Fig. 2:**
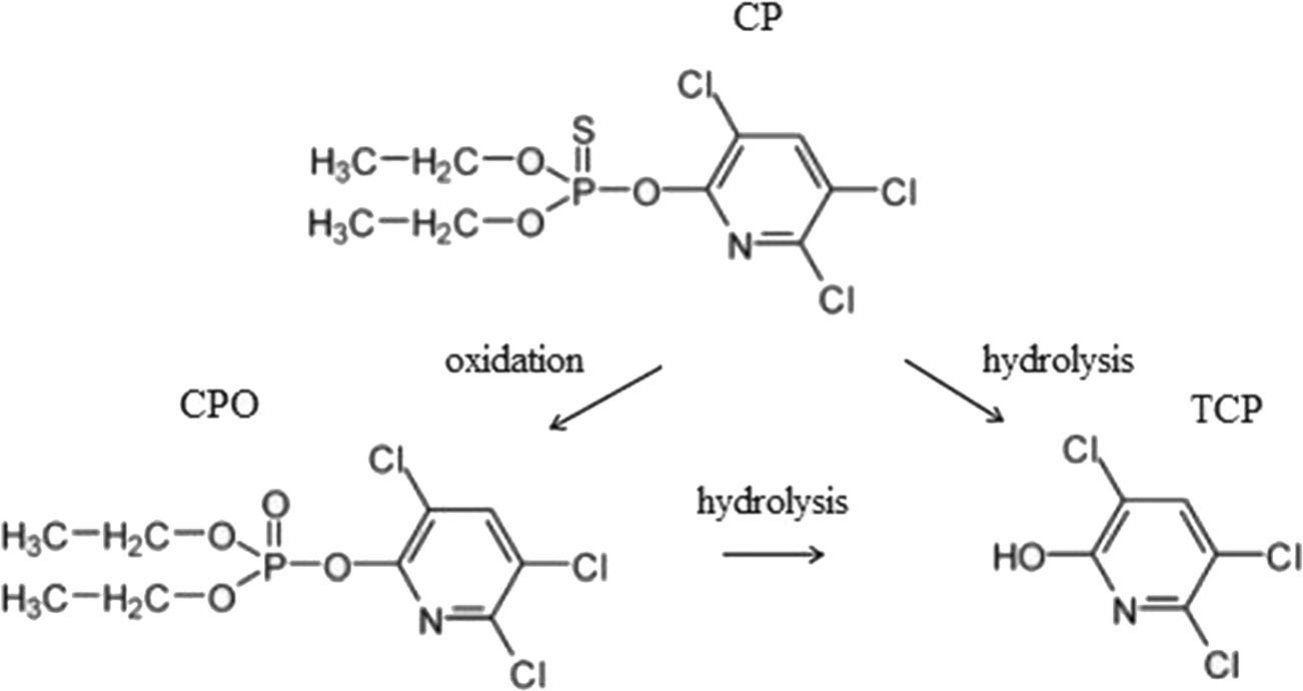
Proposed pathway

The cell potential did not show appreciable variation during the process. Hence, the conductive-diamond layer was not affected by significant deterioration or passivation phenomena. GC-MS analysis confirmed the effectiveness of the treatment, as well as the presence of oxidative and hydrolytic pathways leading to the formation of small amounts of metabolites (Fig. 2). The P = S bond of chlorpyrifos is inclined to be oxidized to P = O bond by the free radicals, leading to the formation of OCP. TCP formation has been ascribed to both CP and CPO hydrolysis (Duirk et al. 2008; Hua et al. 1995).

The same intermediates were detected by liquid chromatography–time of flight-mass spectrometry (LC–TOFMS) in the electrochemical degradation of CP carried out with BDD anode; both compounds were still found after 720 min of treatment (Robles-Molina et al. 2012).

**Fig. 3:**
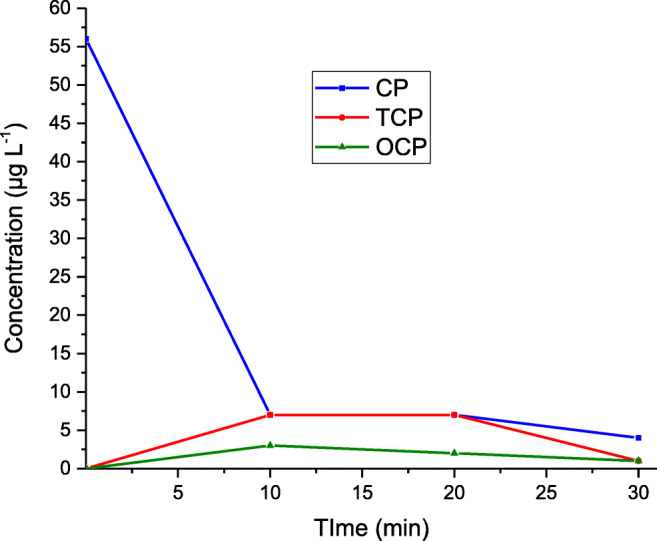
Anodic oxidation at 100 mA. Sonication: Off

The removal obtained by anodic oxidation at 0.1 A is shown in Fig. 3. More than 87% of CP was degraded in the first 10 min. The removal was accompanied by the generation of 7 µg L^− 1^ of TCP and 3 µg L^− 1^ of OCP. At the end of the treatment (CP concentration: 4 µg L^− 1^) the concentration of the metabolites was reduced to 1 µg L^− 1^.

The degradation rate observed here by the use of an SPE to overcome the low conductivity of the treated solution is significantly higher than those previously reported. A 73% removal of the initial COD (450 mg L^− 1^) was attained after 6 h of electrodegradation on BDD anode at 30 °C in acidic medium by Samet and co-workers, which also reached a complete COD abatement by raising the temperature to 70 °C. H_2_SO_4_ and NaOH were employed as conductive electrolytes and for pH adjustment (Samet et al. 2010). More recently, Robles-Molina and co-workers attained a complete CP removal (initial CP concentration: 0.1-1 mg L^− 1^) within 720 min of anodic oxidation on BDD anode and stainless steel (AISI 304) cathode, using 5 g L^− 1^ of sodium sulphate as supporting electrolyte.

Increasing the current intensity from 0.1 to 0.5 A did not increase the final removal (Table 2), although it is likely to be accomplished in a shorter time, as demonstrated by the anodic oxidation of the herbicide terbuthylazine (Tasca et al. 2019a). The effectiveness of ultrasound-irradiation on chlorpyrifos degradation has already been demonstrated in a previous investigation (Zhang et al. 2011), where the coupling with other advanced oxidation processes has been suggested due to lack of mineralization. Here, the effect of sonication on the removal efficiency was not appreciable, while the introduction of this technique significantly enhanced the energy demand, as shown by Table 3. The energy requirements of the vertical stirrer were not taken into account. However, this input must be considered at a major scale, especially when the mass transport near the anode becomes fundamental (i.e.: at low pollutant concentration).

**Table 2:** CP, TCP and OCP concentrations. Specific removal is referred to the sum of CP, TCP and OCP

Electric current (mA)	Sonication	time (min)	CP (µg L^− 1^)	TCP (µg L^− 1^)	OCP (µg L^− 1^)	Removal (%)
100		0	56 ± 0.5	0 ± 0	0 ± 0	-
	Off	10	7 ± 0	7 ± 0	3 ± 0	69.64
		20	7 ± 0.5	7 ± 0.5	2 ± 0	71.43
		30	4 ± 0.5	1 ± 0.5	1 ± 0	89.28
100	On	0	56 ± 0.5	0 ± 0	0 ± 0	-
		10	7 ± 0.5	7 ± 0.5	3 ± 0.5	69.64
		20	7 ± 0.5	7 ± 0.5	2 ± 0.5	71.42
		30	5 ± 0	2 ± 0.5	1 ± 0.5	85.71
500	Off	0	56 ± 0.5	0 ± 0	0 ± 0	-
		30	5 ± 0.5	1 ± 0	1 ± 0.5	87.5
500	On	0	56 ± 0.5	0 ± 0	0 ± 0	-
		30	5 ± 0	1 ± 0	2 ± 0.5	85.71

**Table 3:** Specific energy consumption, referred to the sum of CP and related metabolites

Electric current (mA)	Sonication	time (min)	Energy (KWh µg^− 1^ removed)	(KWh m-^3^ treated)
100		10	8.68∙10^− 6^	0.339
	Off	20	1.69∙10^− 5^	0.678
		30	2.03∙10^− 5^	1.017
		10	9.34∙10^− 4^	36.439
100	On	20	1.82∙10^− 3^	72.878
		30	2.28∙10^− 3^	109.35
500	Off	30	7.65∙10^− 5^	3.75
500	On	30	2.33∙10^− 3^	111.75

The initial CP concentration was very low. Hence, mass transport is fundamental. The stirring rate enhances the mass transport of CP and its metabolites to the anode surface region, as confirmed by our previous investigation on the sono-electro degradation of the antibiotic ciprofloxacin (Tasca et al. 2020). The specific energy consumption increases as the pollutant concentration approaches to low values, as the mass-transport limits the oxidative pathway and the anodic reaction is likely to be mainly the oxygen evolution:

8 $$2\text{H}_2\text{O}\rightarrow\text{O}_2+4\text{H}^++4\text{e}^-$$

The same phenomenon was observed when high current intensity is applied (Klidi et al. 2019; Tasca et al. 2020), due to the high rate of generation of hydroxyl radicals. Hydrogen peroxide and hydroperoxyl radical are liked to be formed from unreacted •OH (Eqs. 9 and 10); given their reduced oxidation power if compared with hydroxyl radicals (Matin et al. 2018; Sabeti et al. 2017), a reduction of the mineralization efficiency is expected (Kaur et al. 2019), notwithstanding the generation of hydroxyl radicals by further reaction of H_2_O_2_ with O_2_ and e^−^ (Yousefzadeh et al. 2018).

9 $$2\bullet\text{OH}\rightarrow\text{H}_2\text{O}_2$$

10 $$\text{H}_2\text{O}_2+\bullet\text{OH}\rightarrow\text{HO}_2\bullet+\text{H}_2\text{O}.$$

Robles-Molina and co-workers confirmed an increasing efficiency with the organic load and a decreasing performance with the current density (Robles-Molina et al. 2012). The theoretical current charge passed estimated to reach a complete removal of 0.1 mg L^− 1^ of CP and assuming the direct mineralization to carbon dioxide (2.981 × 10 − 4 Ah L^− 1^) was found significantly lower than the experimental findings. This support the hypothesis that the electrochemical degradation of the pesticide is a uniquely mass-transfer controlled process, with no significant extension of the oxidation process from the anode surface to the bulk of the solution by inorganic oxidants.

The pollutant removal due to ultrasound irradiation depends on the proximity of the target molecules to the cavitation bubbles. Hydrophobic compounds tend to accumulate at the cavitation bubble interface, where hydroxyl radicals are at high concentration. Here, the increased amount of hydroxyl radicals generated by sonication may lead only to parasite reactions, due to mass transport limitations. Hence, the effect of ultrasound irradiation was not appreciable, notwithstanding the proven efficiency of this technique as a stand-alone treatment for CP degradation (Agarwal et al. 2016). This hypothesis was also supported by the higher removal obtained in a previous study by ultrasound irradiation at low current when the stirring rate was increased (Tasca et al. 2020).

Higher stirring rate would enhance the efficiency of both anodic oxidation and ultrasound irradiation, due to the increase of the mass transfer, while a temperature increase would favor the desired reactions only within a certain range. Indeed, by enhancing the temperature surface tension is reduced, as well as the threshold intensity at which cavitation occurs (Cañizares et al. 2006). On the other hand, further temperature increase would cause more vapor of the target compound and water to being diffused into the cavitation bubble, improving the resistance of the bubble to the inward motion during the collapse and weakening the cavitational collapse (Zhang et al. 2011). Concerning the effect of pH, no significant differences were observed for CP removal by sonication at (130 kHz, 20 min) at pH values of 4, 7 and 9 (Agarwal et al. 2016).

Anodic oxidation at current intensity > 0.1 A or coupled with ultrasound irradiation may be recommended only when higher pollutant concentration is expected, or higher stirring rate is applied. Further enhancement of these two parameters may demand higher frequencies and power of sonication. Indeed, the highest sonochemical production of •OH is expected at frequencies of ~ 300 kHz (Torres-Palma and Serna-Galvis 2018), while an increase of the sonication power enhances the ultrasonic energy transmitted into the treated media and thus the cavitation activity, increasing the number of collapsing bubbles and leading to a high concentration of free radicals (Dükkanci and Gündüz 2006). A slight enhancement of CP degradation through ultrasound irradiation was recently recorded by Agarwal and co-workers, by increasing sonication frequency and power from 35 to 130 kHz and from 300 to 500 W, respectively (Agarwal et al. 2016). Moreover, sonication efficiency can be enhanced by the use of ultrasonic probe-type devices in place of ultrasonic baths, due to a focused and uniform ultrasonic power input, as cavitation occurs non-conformable and uncontrollably in an ultrasonic bath. The effect of the input parameters on the desired response as been successfully assessed by recent investigations on water remediation (Aslani et al. 2017, 2016; Tasca et al. 2020). Hence, Response Surface Methodology (RSM) is suggested as further step to define the feasibility of sonication coupled with anodic oxidation.

Furthermore, the application of anodic oxidation and ultrasound irradiation to natural waters is expected to require an increased demand of radicals production, due to the scavenging effect of humic acids and ions. The reaction of HCO^3−^ with hydroxyl radicals and Cl∙ is expecting to generate CO^3−^∙, which has a low oxidation potential if compared with •OH. Nitrate ions can also interact with hydroxyl radicals and being reduced to NO^2−^. The effect of chloride ions is controversial, as they may react with •OH and Cl∙, giving to weak oxidizing capability radicals, which in turn would be decomposed into •OH and Cl∙ again with a high rate constant (Deng et al. 2019).

## Conclusions

This study demonstrated that chlorpyrifos can be effectively removed from water by anodic oxidation and ultrasonic irradiation, even at concentration < 0.1 mg L^− 1^. Based on GC–MS analysis, two degradation products due to oxidative and hydrolytic pathways were detected. Electrolysis carried out by a Nb/BDD anode, a Ti/RuO_2_ mesh cathode, and a Nafion® N324 ion exchange membrane resulted in more than 87% of CP removal within the first 10 min of treatment. The degradation of CP was accompanied by the generation of a small amount of by-products, further reduced in the following 20 min. Sonication provided at 40 kHz did not enhance the performance of the system, while highly increased the energy demand, as mass transport limitations hindered the removal potential of hydroxyl radicals. Coupling anodic oxidation with sonication is recommended only when high contaminant load is to be treated, or when high stirring rate is provided.

The SPE technique allows the successful processing of low conductivity solutions. Anodic oxidation can assist or replace the disinfection phase of water treatment facilities, with no additional sludge production. Further research is encouraged, with the aim to identify the threshold concentration and the associated stirring rate which would define the economic feasibility of ultrasound addition to anodic oxidation-based systems.
